# Procedures Relative to the Single Care of Insane Persons

**Published:** 1900-07-28

**Authors:** F. St. John Bullen


### Hospital Clinics and Medical Progress.

### "PROCEDURES RELATIVE TO THE SINGLE CARE OF INSANE PERSONS."

By F. St. John Bullen, M.R.C.S.

(*Continued from page* 268.)

It will be seen that the medical man in charge of an insane patient has no functions other than those allotted to a lay person in similar capacity, with these exceptions: He may prescribe ordinary medical treatment, and, with the special sanction of the Commissioners, he may make some of the fortnightly notes instead of the medical attendant, thus rendering the visits of the latter less necessarily frequent. In the case of a Chancery patient, the medical visit once a fortnight is not required. He should, however, be acquainted not merely with his own particular duties, but also with those of the medical attendant, who cannot be expected to keep them in mind. These procedures include supervision of all documents in connection with the patient, and the making of certain reports to the Commissioners (or Chancery visitors) which deal with the admission, removal, discharge or death, escape and recapture, restraint, regulation of correspondence, visits, and some other matters. These may be considered in detail. The examination of the certificates and other forms authorising the detention of the patient needs to be carefully conducted. Immaterial errors, that is to say, defects in the order or certificate not sufficiently grave to forbid the admission of the patient, can be amended within 14 days, with the sanction of the Commissioners and of the Justice who made the order. The Commissioners will indicate the faulty portion of the form, and the person or persons responsible for the error must be directed to amend this and obtain the consent of the Justice. Material errors may involve the discharge of the patient and fresh procedures for re-certification, besides laying the person detaining the patient open to a charge of false imprisonment. Copies of the several documents received with the patient have to be made, and, together with a notice of admission containing full particulars of the residence and title of the proprietor and person in charge, must be sent to the Commissioners not later than the end of the day following that on which the patient was received. These copies must be certified under the hand of the person in charge. He must see in the case of an urgency order that the additional forms required are being prepared, and will be delivered to him within seven days, or that he receives a written assurance from the petitioner that a petition to a justice is actually pending, or the patient must be discharged at the end of seven days after reception, excluding the day of date of the order. The documents upon which the patient is admitted should be copied into the Medical Journal, or affixed thereto. Within three days of reception he must see that the medical attendant has thoroughly examined the patient, and has entered full particulars of the mental and bodily condition in the Medical Journal and signed the same; and that between the third and seventh day he prepares and forwards to the Commissioners a report of the mental and bodily condition of the patient, a copy of which he enters in the Journal. The frequency of the visits to be made by the medical attendant is determinable by the Commissioners; and, as already stated, they may not require an invariable fortnightly visit to be paid. The next report concerning the condition of mind and body of the patient has to be sent not later than two days before the expiration of a month from the time of admission, but this procedure is not needed in the case of a person removed from other care, or if a Chancery patient. In the ordinary course of events no further reports need to be sent to the Commissioners, except the two following ones—one between January 10th and 17th annually, and another one year from the date of the reception order, this latter being repeated at gradually increasing intervals. The continuation orders are vouchers for the mental and bodily state of the patient. The annual report in the case of a Chancery patient is returned not less than two days before May 1st of the year ensuing on the patient's reception. There are some contingencies that may arise, and which need to be more or less rapidly dealt with. The patient may escape, and, provided there seems no chance of immediate recapture, it will be requisite to communicate at once with the petitioner and any persons to whom the lunatic may make his way, and to take all usual measures for resuming control over a person illegally at large. Care should be taken to have all necessary proofs of authority forthcoming, either in person or by deputy, and to inform the Commissioners, or the Chancery visitors and the committee, within three days.

The recapture must be made before 14 days have elapsed, or fresh procedures for certification must be undertaken. The patient may, however, escape into Scotland or Ireland, in which case, although means may be taken to track his whereabouts, no recapture can be made until the Commissioners have allowed application to be made to the Justice in the place whence the lunatic has escaped, and from whom authority can be obtained for the recapture. Such authority will only be effective after counter-signature by Sheriff in Scotland, or Justice in Ireland. Within three days of repossession of the patient notification needs to be sent to the Commissioners. Supposing it be desirable to give the patient a holiday, or to allow out on trial, the Commissioners' consent must be obtained, and the application must be made by the petitioner or person paying for the patient; a certificate recommending the change must be procured from the medical attendant, and forwarded with the application. It must be stated where, and for how long it is proposed to remove the patient, and that he will continue to be under effective care and control. In the case of a patient "so found," it falls to the committee to acquaint the Chancery visitors. The person having charge of the lunatic may, however, remove to another residence in England without doing more than give seven days' notice in writing to the Commissioners and to the petitioner or payer. This removal cannot be made in the case of a Chancery patient without the consent of the latter's committee. If the petitioner wish to place the patient under the charge of another person or in an institution he must obtain an order of removal from the Commissioners. Here the medical man in charge has only two duties to perform, which are to furnish certified copies of the documents received with the patient and to send a notice of the transfer to the Commissioners (or Chancery visitors as the case may be) within three days. He must not relinquish charge of the patient unless he receives and retains an order of removal.

